# Substitution analyses of foods with varying fat quality and the associations with all-cause mortality and impact of the FADS-1 genotype in elderly men

**DOI:** 10.1007/s00394-023-03249-y

**Published:** 2023-09-20

**Authors:** Michael Fridén, Erika Olsson, Lars Lind, Fredrik Rosqvist, Ulf Risérus

**Affiliations:** 1 Department of Public Health and Caring Sciences, Clinical Nutrition and Metabolism, Uppsala University, Uppsala, Sweden; 2 Department of Surgical Sciences, Medical Epidemiology, Uppsala University, Uppsala, Sweden; 3 Department of Medical Sciences, Clinical Epidemiology, Uppsala University, Uppsala, Sweden

**Keywords:** Food substitutions, Dietary fat quality, FADS, Mortality, CVD

## Abstract

**Purpose:**

To investigate associations between substitutions of foods varying in fat quality and all-cause mortality in elderly Swedish men and to examine effect measure modification by a gene involved in fatty acid desaturation (rs174550 FADS1).

**Methods:**

Using Cox-regression models in the ULSAM cohort (n = 1133 men aged 71), we aimed to investigate; (1) Associations between the substitution of a nutrient or food for another on all-cause mortality (primary outcome) and CVD (secondary outcome) and (2) Associations between the addition of various fat-rich foods to the habitual diet and all-cause mortality and CVD. Subgroup analyses based on the rs174550 FADS1 genotype were conducted.

**Results:**

Over a mean follow-up of 11.6–13.7 years, n = 774 died and n = 494 developed CVD, respectively. No clear associations were observed for the vast majority of substitution nor addition models. Adding saturated fatty acids (SFA) on top of the habitual diet was however associated with an increased risk of mortality in men with the CT/CC-genotype [HR (95% CI) 1.44 (1.05, 1.97)]. Post-hoc analyses showed an inverse association of substituting SFA with carbohydrates [HR (95% CI) 0.79 (0.65, 0.97)], which was somewhat stronger in men with the CT/CC-genotype compared to men carrying the TT-genotype.

**Conclusions:**

Few associations were observed between diet and all-cause mortality and CVD in this population. However, substituting SFA with carbohydrates was associated with lower mortality in post-hoc analyses and adding SFA to the habitual diet increased mortality in men with the CT/CC-genotype. The latter observation is novel and warrants further investigation in larger cohort studies including women.

**Supplementary Information:**

The online version contains supplementary material available at 10.1007/s00394-023-03249-y.

## Background

Based on both randomized trials and prospective cohort studies, global dietary recommendations, including those from the Nordic countries (Nordic Nutrition Recommendations (NNR)), highlight the importance of replacing saturated fatty acids (SFA) with mono (MUFA)- and polyunsaturated fatty acids (PUFA) in the diet [1]. However, longitudinal data on hard outcomes such as all-cause mortality and cardiovascular disease (CVD) with clearly defined food substitutions whereby one food item rich in SFA is replaced by another food item rich in PUFA in an elderly Nordic population, are limited. Whether single-food replacements differing in dietary fat quality later in life may be sufficient to counteract other age-related biological processes, is unclear. Several meta-analyses of observational studies have been conducted to investigate the association between intake of SFA and all-cause mortality and CVD in primarily younger and middle-aged populations, some of which have concluded that SFA is not associated with either outcome [2–4]. However, methodological limitations including adjustment for causal intermediates such as blood lipids and blood pressure might have led to biased estimates, thereby attenuating these associations. Furthermore, since many previous observational studies have adjusted for total energy intake without specifying any isocaloric replacement nutrient or food, an unspecified substitution effect of increasing SFA at the expense of a weighted average of all other energy-providing foods or nutrients is estimated [5–7]. The obtained risk estimate is therefore strongly reliant on the population-specific background distribution of the diet, making it somewhat difficult to compare findings between populations and even harder to interpret pooled estimates from meta-analyses [5].

Randomized controlled trials (RCT) have generally shown a benefit of consuming less compared with more SFA on composite CVD events [8, 9]. However, these studies have primarily been conducted in middle-aged individuals and are limited by the fact that different comparator foods or diets have been used and that they are underpowered to detect effects on all-cause mortality [8]. Clearly specifying the replacement nutrient(s) and/or food(s) in both observational and randomized trials may help to overcome the issue of comparability raised above [10, 11].

Accumulating data have shown that variation in the FADS 1–3 gene cluster can impact the metabolism of dietary PUFA, and especially the FADS1 gene has been linked to several cardiometabolic disease traits, and may be a potential modifier of fatty acid intake and mortality risk [12]. Recent intervention studies of PUFA intake on cardiometabolic risk markers have demonstrated effect heterogeneity by a single nucleotide polymorphism (SNP) in a gene responsible for desaturating PUFA (rs174550 in the FADS1 gene) [13, 14]. Lankinen, et al. showed that when individuals with the TT-genotype in the rs174550 FADS1 gene consumed a PUFA-rich diet, they responded with lower levels of plasma C-reactive protein but higher levels of adipose tissue inflammatory markers, compared to individuals with the CC-genotype [13–15]. Longitudinal studies investigating gene-diet interactions of substituting foods rich in SFA with foods rich in PUFA on hard outcomes such as all-cause mortality and CVD are however limited, but important to examine in an era focused on personalized medicine/nutrition. Our primary aim of this study was therefore to examine associations between isocaloric substitutions of SFA and SFA-rich foods with PUFA and PUFA-rich foods on all-cause mortality and CVD in an elderly Swedish population as well as to investigate potential effect measure modification by the rs174550 FADS1 genotype. Our secondary aim was to examine associations between adding a certain food or nutrient on top of the habitual diet on all-cause mortality and CVD in the full population as well as between stratum of the rs174550 FADS1 genotype.

## Methods

### Study population

The Uppsala Longitudinal Study of Adult Men (ULSAM) is a population-based clinical cohort study initiated in 1970, whereby n = 2322 50-year old men were recruited from the general population to investigate risk factors for CVD [16]. In 1991–1995, n = 1221 71-year old men from the original cohort were included for a follow-up investigation. Of these, n = 1138 had data on dietary intake. Dietary habits were assessed for the first time in 1991–1995. After exclusion of energy misreporters (< 800 kcal/day and > 4200 kcal/day), n = 1133 remained, constituting our main study population. Furthermore, of these n = 1133, n = 1084, had data on the rs174550 FADS1 genotype for the stratified analyses. For the secondary outcome (CVD), n = 162 with prevalent CVD at baseline were excluded (Supplementary Fig. 1, Online Resource 1). This study was conducted in accordance with the guidelines laid down in the Declaration of Helsinki and was ethically approved by the Regional Ethical Review Board at Uppsala University (Dnr 251/90 and Dnr 2013/350). All participants gave their written informed consent prior to inclusion.

### Exposure assessment (diet)

For the baseline measures in 1991–1995, habitual diet was assessed using an optically readable form of a seven day dietary record with the use of a pre-coded menu book from the Swedish National Food Agency (SNFA, version 1990) [17]. Intakes of different foods were reported in household measures or in predefined portion sizes. Participants were also able to report other foods not specified in the menu book in free text. The menu book was validated against an open ended seven day weighed food record in a subsample of the cohort [18]. The seven day dietary assessment method has furthermore been validated against biomarkers in middle-aged individuals [19]. Dietary intake was analyzed with the help of a food composition database from the Swedish National Food Agency (SNFA, version 1990)) incorporated into a commercially available software.

Food items from the 7 day dietary records were classified in the following 10 food categories: red and processed red meat (hereafter termed meat), unprocessed fish (hereafter termed fish), butter and butter-based spreads (hereafter termed butter), margarine and vegetable oils (hereafter termed vegetable oils), fruits and vegetables (including legumes) (hereafter termed F&V), alcoholic beverages, non-alcoholic beverages (excluding water, tea and coffee), total dairy (hereafter termed dairy), fatty snacks and pastries and other foods (e.g. cereals, mayonnaise and white meat). All foods were expressed in 100 kcal. Blank responses were recorded as zero. Nutrients were classified in the following six categories: total carbohydrates, total protein, alcohol, SFA, MUFA and PUFA. All nutrients were expressed in 100 kcal.

### Genotyping

FADS1 (rs174550) was genotyped after DNA extraction from blood samples using the Illumina Human Omni2.5M. Due to low number of participants homozygous for the CC-genotype, CC and CT were combined, thereby creating two strata (TT and CT/CC).

### Substitution and addition modelling

Substitution analyses were performed using the leave-one-out method with all foods and nutrients expressed in kcal [6, 20, 21]. Total energy intake was included as a composite variable to account for confounding by common determinants of dietary intake [5, 22]. Substitutions of interest were meat with fish, butter with vegetable oils, SFA with PUFA as well as meat with F&V, butter with F&V and SFA with carbohydrates, the latter three being post-hoc substitutions. The former a priori determined substitutions were included due to two main reasons: (1) food and nutrient substitutions had to reflect real-life everyday replacements and (2) foods had to reflect a significant proportion of SFA and PUFA in the Swedish diet. The latter is the main reason why red and processed red meat was not substituted for white meat. Due to low intakes of nuts and seeds in this population, prespecified substitutions such as nuts and seeds with dairy was not feasible. The leave-one-out method has been shown to perform as good as the energy partition method when the statistical modelling involves substituting just one nutrient or food for another [21]. The leave-one-out model mimics an RCT by which the intervention group would receive x amount of kcal from food 1 while the comparator group would receive x amount of kcal from food 2, holding all other foods constant between the groups, thereby making them isocaloric. Examples from two statistical modelling approaches (one for nutrients and one for foods) using this method are provided below.

1. Log(h(t; x)) of replacing SFA with PUFA = log(h_0_(t)) + β_1PUFA_ + β_2MUFA_ + β_3Carbohydrates_ + β_4Protein_ + β_5Alcohol_ + β_6Totalenergyintake +_ β_7Confounders_
2. Log(h(t; x)) of replacing red meat with fish = log(h_0_(t)) + β_1Fish_ + β_2Butter_ + β_3Vegetableoils_ + β_4Fattysnacksandpastries_ + β_5Dairy_ + β_6Alcoholicbeverages_ + β_7Non-alcoholicbeverages_ + β_8F&V_ + β_9Otherfoods_ + β_10Totalenergyintake_ + β_11Confounders_

Statistically modelling adding a nutrient or food on top of the habitual diet was performed using the all-components model suggested by Tomova et al. [5]. Additions of interest were all foods and nutrients included in the primary analysis (except for carbohydrates and F&V). The all-components model mimics an RCT by which the intervention group would receive x amount of extra kcal from food 1 while the comparator group would continue with their habitual diet, thereby making them non-isocaloric. Although mediated by an increase in energy intake, this model is the only model that can estimate total effects from diet, in line with RCTs such as the omega-3 supplementation trials or a recent study investigating the effect of adding one avocado per day to the background diet on visceral adipose tissue [23, 24]. Due to the non-isocaloric condition that is inherently imposed, this model was ranked secondary to the leave-one-out model with regards to real-life practical relevance. Examples from two statistical modelling approaches (one for nutrients and one for foods) using this method are provided below.

1. Log(h(t; x)) of adding PUFA = log(h_0_(t)) + β_1PUFA_ + β_2MUFA_ + β_3SFA_ + β_4Carbohydrates_ + β_5Protein_ + β_6Alcohol_ + β_7Confounders_
2. Log(h(t; x)) of adding fish = log(h_0_(t)) + β_1Fish_ + β_2Meat_ + β_3Butter_ + β_4Vegetableoils_ + β_5Fattysnacksandpastries_ + β_6Dairy_ + β_7Alcoholicbeverages_ + β_8Non-alcoholicbeverages_ + β_9F&V_ + β_10Otherfoods_ + β_11Totalenergyintake_ + β_12Confounders_

All substitutions and additions were performed with 100 kcal as the unit of exposure, corresponding to about 6 E% in this population reporting a median total energy intake of 1712 (IQR 584) kcal. The main reason for choosing kcal instead of E% as the unit of exposure is the somewhat obscure estimand obtained when including ratio variables such as E% in regression models [5].

### Confounders

Confounders were identified using directed acyclic graphs (DAGs) with subject matter knowledge and the use of the DAGitty tool (Dagitty.net) [25]. To estimate the joint effect of substituting SFA-rich foods with PUFA-rich foods, a minimally sufficient adjustment set of variables were identified: age (continuous), current smoking (yes/no), education (elementary school/folk high school/upper secondary school or equivalent/university or equivalent), family history of diabetes (yes/no), family history of CVD (yes/no), physical activity (over/under 3 h of moderate physical activity per day), sleep (difficulties/no difficulties getting to sleep at night), stress (have/have not experienced stress at home or at work the past 5 years), total energy intake (kcal) and all foods/nutrients (kcal) except for the one to be substituted (Supplementary Fig. 2, Online Resource 1). All covariates were self-reported. To estimate the total effect of adding SFA/PUFA-rich foods on top of the habitual diet, a similar adjustment set was identified (except for total energy intake and the food/nutrient that was left out in the leave-one-out model) (Supplementary Fig. 2, Online Resource 1).

### Outcome ascertainment (all-cause mortality and CVD)

Deaths from all causes were retrieved from the Cause of Death Registry and was the primary outcome of the study. CVD was composed of primary CVD (myocardial infarction (ICD-8: 410, ICD-9: 310, ICD-10: I20), ischemic stroke (ICD-8: 431, 433–436, ICD-9: 431, 433–436, ICD-10: I63-I66) and heart failure (ICD-8: 427.00, 427.10, 428.99, ICD-9: 428, ICD-10: I50, I11.0) and was identified through linkage with the Swedish National Patient and Cause of Death Registries. The accuracy of myocardial infarction and stroke have been deemed to be high in Swedish registries [26]. However, as heart failure has shown lower validity, additional chart review based validation was performed, as previously described [27]. Those with prevalent CVD at baseline were excluded for the analyses of incident CVD. CVD was determined the secondary outcome.

### Statistical analyses

Our primary aim of this study was to investigate the associations between substituting SFA-rich foods with PUFA-rich foods and all-cause mortality and CVD, respectively. Multivariable Cox proportional hazard regression models were used to estimate hazard ratios (HR) with corresponding 95% CI, with time since baseline as the underlying time scale. Time at risk for all-cause mortality was calculated from the date of first baseline visit (1991–1995) until the date of death or administrative end of follow-up (December 31, 2011), whichever occurred first. For CVD, time at risk was calculated from the date of first baseline visit (1991–1995) until the date of CVD diagnosis, death or administrative end of follow-up (December 31, 2011), whichever occurred first. Missing data on confounders (2% for family history of T2D and family history of CVD, 3% for physical activity, sleep and stress, and 4% for smoking) were imputed using multiple imputation (n = 5 imputations) to account for potential selection bias, assuming the data was missing at random (MAR). Energy misreporters (n = 5) were excluded to account for potential measurement bias. Similar models were used for the secondary aim of this study of investigating associations of adding SFA/PUFA-rich foods on all-cause mortality and CVD. Effect measure modification by the rs174550 FADS1 genotype on the multiplicative scale was examined by stratified Cox regression analyses. The assumption of proportional hazards was checked using Schoenfeld residual plots.

Post-hoc analyses included examining the associations between substituting SFA and SFA-rich foods with carbohydrates and carbohydrate-rich foods and all-cause mortality and CVD. These analyses were additionally performed since dietary recommendations also highlight that saturated fat as a whole should be limited, but without clearly specifying the replacement nutrient or food. The carbohydrate-rich food used in the food substitution models was F&V.

To investigate the robustness of our results, a couple of sensitivity analyses were performed including (1) complete-case analyses (to address the question of selection bias) and (2) excluding the first 2 years of follow-up (to address the question of reverse causation). All analyses were carried out in IBM SPSS Statistics version 28.0.1.0 (142).

## Results

Baseline characteristics for the primary (all-cause mortality) and secondary population (CVD) are presented in Table 1. Study participants were all men and had a median age of 71 (IQR 0.80) at baseline. Over half (58.9–59.8% for all-cause mortality and CVD, respectively) reported that they engaged in moderate physical activity more than 3 h per week, 14.2–14.5% (for all-cause mortality and CVD, respectively) were current smokers and 14.7–15.0% (for all-cause mortality and CVD, respectively) had a university degree. Self-reported median daily energy intake was 1712 (IQR 584) kcal for the primary population and 1718 (IQR 592) kcal for the secondary population. For the rs174550 FADS1 genotype, 40.4% were homozygous for the TT-variant in both populations. Baseline dietary intake stratified by the rs174550 FADS1 genotype are presented in Supplementary Table 1, online resource 1. Over a mean follow-up of 13.7 years (maximum 20.4 years and 15,498 person-years) for all-cause mortality and 11.6 years (maximum 20.4 years and 11,275 person-years) for CVD, n = 774 cases of all-cause mortality was captured and n = 494 developed CVD.

**Table 1:** Population characteristics according to the event studied: all-cause mortality and CVD

	All-cause mortality (n = 1133)	CVD (n = 971)
Sex (n (%) men)	1133 (100)	971 (100)
Age (years)	71 (0.80)	71 (0.80)
University education (n (%))	167 (14.7)	146 (15.0)
Current smokers (n (%))	161 (14.2)	141 (14.5)
Physical activity (n (%) of 3 h/week of MPA)	667 (58.9)	581 (59.8)
Experience of stress (n (%))	37 (3.3)	28 (2.9)
Difficulties sleeping at night (n (%))	121 (10.7)	97 (10.0)
Family history of T2D (n (%))	167 (14.7)	144 (14.8)
Family history of CVD (n (%))	669 (59.0)	559 (57.6)
rs174550 FADS1 (n (%) of TT/TC and CC)	458 (40.4)/626 (55.3)	393 (40.4)/539 (55.5)
Total energy intake (kcal/d)	1712 (584)	1718 (592)
Butter and butter-based spreads (kcal/d)	63 (102)	63 (108)
Margarine and vegetable oils (kcal/d)	0 (61)	0 (61)
Unprocessed fish (kcal/d)	0 (6)	0 (6)
Red and processed red meat (kcal/d)	133 (94)	134 (94)
SFA (kcal/day)	255 (123)	257 (124)
PUFA (kcal/day)	84 (40)	86 (39)

Data are presented in medians (IQR) for continuous variables and counts and percentages for categorical variables

For descriptive statistics in Table 1, physical activity is based on n = 1103 for all-cause mortality and n = 945 for CVD; rs145550 FADS1 on n = 1084 for all-cause mortality and n = 932 for CVD; current smokers on n = 1083 for all-cause mortality and n = 931 for CVD; family history of T2D on n = 1111 for all-cause mortality and n = 952 for CVD; family history of CVD on n = 1114 for all-cause mortality and n = 955 for CVD; difficulties sleeping at night on n = 1102 for all-cause mortality and n = 944 for CVD; experience of stress on n = 1099 for all-cause mortality and n = 943 for CVD

*CVD* Cardiovascular disease, *FADS1* fatty acid desaturase enzyme 1, *MPA* moderate physical activity, *PUFA* polyunsaturated fatty acids, *SFA* saturated fatty acids, *T2D* Type-2 diabetes

### Substitution analyses (leave-one-out model)

No associations were observed for substituting SFA with PUFA or meat with fish on either all-cause mortality or CVD (Table 2 for the full population and Table 3 for the stratified population). Substituting butter with vegetable oils indicated an inverse association with all-cause mortality [HR 0.93 (95% CI 0.84, 1.02)) and CVD (HR 0.89 (95% CI 0.79, 1.01)] (Table 2). Substituting SFA with carbohydrates was associated with a 21% decreased risk of all-cause mortality [HR 0.79 (95% CI 0.65, 0.97)] in the full population (Table 2) and with a 26% decreased risk of all-cause mortality in participants with the CT/CC-genotype [HR 0.74 (95% CI 0.56, 0.98)] in post-hoc analyses (Table 3). For the TT-genotype the HR was 0.79 (95% CI 0.57, 1.11) (Table 3).

**Table 2:** Associations between food and nutrient substitutions and additions and all-cause mortality and CVD

	All-cause mortality	CVD
*Leave-one-out model*		
SFA with PUFA	1.27 (0.86, 1.88)	1.54 (0.93, 2.54)
Butter with oils	0.93 (0.84, 1.02)	0.89 (0.79, 1.01)
Meat with fish	1.29 (0.84, 1.98)	1.30 (0.76, 2.24)
SFA with carbohydrates	0.79 (0.65, 0.97)	0.93 (0.71, 1.20)
Butter with F&V	0.89 (0.77, 1.03)	0.93 (0.78, 1.11)
Meat with F&V	1.00 (0.86, 1.15)	1.04 (0.87, 1.24)
*The all-components model*		
SFA	1.18 (0.93, 1.49)	1.01 (0.75, 1.35)
PUFA	1.53 (0.94, 2.48)	1.55 (0.85, 2.84)
Butter	1.11 (0.99, 1.23)	1.07 (0.93, 1.22)
Oils	1.03 (0.96, 1.11)	0.95 (0.87, 1.05)
Fish	1.27 (0.83, 1.94)	1.24 (0.73, 2.12)
Meat	0.99 (0.89, 1.09)	0.95 (0.84, 1.08)

Data are presented as hazard ratios with 95% confidence intervals for all-cause mortality (n = 1133) and CVD (n = 971)

Both models are adjusted for age, education, smoking, stress, sleep, family history of CVD, family history of type-2 diabetes and physical activity. The leave-one-out model is furthermore adjusted for total energy intake and includes all nutrients or foods except for the one to be substituted. The all-components model includes all nutrients or foods. All substitutions and additions are modelled in the unit of 100 kcal

Number of cases = 774 for all-cause mortality and 494 for CVD

**Table 3:** Associations between food and nutrient substitutions and additions and all-cause mortality and CVD, stratified by rs174550 FADS1-genotype

	All-cause mortality		CVD	
	TT	CT/CC	TT	CT/CC
*Leave-one-out model*				
SFA with PUFA	1.53 (0.83, 2.81)	0.96 (0.56, 1.66)	1.32 (0.58, 2.99)	1.31 (0.67, 2.55)
Butter with oils	0.88 (0.76, 1.03)	0.99 (0.86, 1.13)	0.90 (0.74, 1.11)	0.97 (0.82, 1.15)
Meat with fish	1.23 (0.61, 2.48)	1.40 (0.78, 2.53)	1.70 (0.72, 3.99)	1.26 (0.58, 2.74)
SFA with carbohydrates	0.79 (0.57, 1.11)	0.74 (0.56, 0.98)	0.89 (0.59, 1.34)	0.89 (0.63, 1.26)
Butter with F&V	0.84 (0.67, 1.06)	0.95 (0.77, 1.18)	0.86 (0.64, 1.16)	1.07 (0.82, 1.39)
Meat with F&V	0.91 (0.73, 1.14)	1.04 (0.85, 1.28)	0.92 (0.69, 1.23)	1.13 (0.88, 1.46)
*The all-components model*				
SFA	0.98 (0.66, 1.44)	1.44 (1.05, 1.97)	0.96 (0.59, 1.56)	1.15 (0.78, 1.71)
PUFA	1.45 (0.67, 3.15)	1.48 (0.76, 2.88)	1.26 (0.46, 3.41)	1.54 (0.69, 3.41)
Butter	1.09 (0.91, 1.31)	1.09 (0.94, 1.27)	1.02 (0.81, 1.27)	1.02 (0.85, 1.23)
Oils	0.97 (0.85, 1.10)	1.08 (0.99, 1.18)	0.92 (0.76, 1.10)	0.99 (0.88, 1.12)
Fish	1.24 (0.64, 2.44)	1.40 (0.78, 2.50)	1.62 (0.71, 3.67)	1.22 (0.57, 2.62)
Meat	1.01 (0.87, 1.18)	1.00 (0.86, 1.16)	0.95 (0.78, 1.17)	0.96 (0.81, 1.15)

Data are presented as hazard ratios with 95% confidence intervals for all-cause mortality (n = 1084) and CVD (n = 932)

Both models are adjusted for age, education, smoking, stress, sleep, family history of CVD, family history of type-2 diabetes and physical activity. The leave-one-out model is furthermore adjusted for total energy intake and includes all nutrients or foods except for the one to be substituted. The all-components model includes all nutrients or foods. All substitutions and additions are modelled in the unit of 100 kcal

Number of cases = 264 (TT) and 350 (CT/CC) for all-cause mortality and 190 (TT) and 286 (CT/CC) for CVD

### Addition analyses (all-components model)

In the all-components model, the HR for SFA intake on all-cause mortality in the full population was 1.18 (95% CI 0.93, 1.49) (Table 2). No associations were observed for intake of PUFA, meat, fish or vegetable oils on either mortality or CVD (Table 2). Butter intake indicated a positive association with mortality [HR 1.11 (95% CI 0.99, 1.23)] in the full population (Table 2) whereas intake of vegetable oils indicated a positive association with mortality [HR 1.08 (95% CI 0.99, 1.18)] in individuals with the CT/CC-genotype (Table 3). SFA intake was associated with a 44% increased risk of all-cause mortality in participants with the CT/CC-genotype [HR 1.44 (95% CI 1.05, 1.97)] but not in participants with TT-genotype [HR 0.98 (95% CI 0.66, 1.44)] (Table 3).

### Sensitivity analyses

Complete-case analyses (Supplementary Table 2 for the full population and Supplementary Table 3 for the stratified population, Online Resource 1) and analyses whereby the first two years of follow-up were excluded (Supplementary Table 4 for the full population and Supplementary Table 5 for the stratified population, Online Resource 1) demonstrated similar associations with both all-cause mortality and CVD as for the main analyses, except for some minor deviations that did not alter the interpretations of the results.

## Discussion

In this prospective cohort study of elderly Swedish men, no clear associations were observed between food and nutrient substitutions nor additions and all-cause mortality and CVD in the whole population, except for the substitution of SFA with carbohydrates that was inversely associated with all-cause mortality. The latter was also shown for men with the combined CT/CC-genotype, but not for the TT-genotype of the FADS1 gene (rs174550). Furthermore, a novel finding was that adding SFA to the habitual diet was associated with all-cause mortality in men with the CT/CC-genotype in the FADS1 gene (rs174550), but not for men carrying the TT-genotype. This association is to our knowledge novel, and might point towards a gene-diet interaction with potential clinical implications.

In line with a newly published meta-analysis of prospective cohort studies of primarily middle-aged populations, we found an inverse association of substituting SFA with carbohydrates on all-cause mortality in a post-hoc analysis [28]. On the contrary, we found no clear associations of substituting SFA-rich foods such as meat or butter with the carbohydrate-rich source F&V on mortality. The inverse association observed on a nutrient level may therefore be due to other isolated or mixed food replacements, warranting further investigation. Lack of associations for the a priori determined food exposures in our study on all-cause mortality are partly in line with a previous Danish cohort study investigating the substitution of 150 g of meat per week with 150 g of fish per week, showing a HR of 0.99 (95% CI 0.97–1.01) [29]. In contrast to our null findings, inverse associations on mortality of substituting SFA with PUFA have been demonstrated in other cohort studies [28, 30, 31]. Few longitudinal studies have however been conducted to investigate the substitution of SFA-rich foods such as butter with PUFA-rich foods such as vegetable oils. Although our findings indicated an inverse association of substituting butter with vegetable oils on mortality [HR 0.93 (95% CI 0.84, 1.02)], the precision of the point estimate was wide, covering an upper CI level of one. Guasch-Ferré et al. showed, using repeated measures of diet, a decreased risk on all-cause mortality when substituting butter with olive oil, but that study was conducted in middle-aged men and women in the U.S with olive oil as the comparator food [32]. The two studies are therefore discrepant on at least three points: different comparator foods, inclusion of both men and women and different age groups. Furthermore, it is possible that trans fatty acid content in some margarines may have contributed to attenuated associations between the substitution of butter with vegetable oil in our population.

Our null findings for CVD are also in line with some observational studies conducted in older individuals [33], but not with others [34]. Steur, et al. showed weaker associations for incident CVD in individuals over 52 compared to those under 52 years of age of substituting carbohydrates with SFA [33]. Interestingly, in the CORDIOPREV study, the Mediterranean diet [defined by higher intakes of unsaturated fat and lower SFA, lower red meat and butter intake and higher intakes of fish, vegetable oils (olive oil) and F&V] compared to a low-fat diet was not associated with CVD when stratified by over 70 years of age [35]. This suggestive age-dependent effect on CVD was also shown in the REDUCE-IT trial where participants received extra energy from 4 g of daily omega-3 fatty acids [23]. These findings may suggest that dietary habits earlier in life may have more profound effects on CVD but potentially also on other cardiometabolic risk factors associated with all-cause mortality. In contrast, the PREDIMED study showed no effect modification by age [36]. Lack of associations in our study on CVD could also be a consequence of lower statistical power, indicated by the wide CI, and should therefore be interpreted with caution. Importantly, these findings do not contradict the body of evidence from both RCTs and observational studies showing a reduction in LDL-cholesterol/apoB containing lipoprotein levels and CVD outcomes in younger and middle-aged populations when substituting SFA and SFA-rich foods with PUFA and PUFA-rich foods [8, 34, 37, 38]. Findings from this study are to be interpreted in the context for which it has been conducted; in elderly men.

Potential effect measure modification by the rs174550 FADS1 genotype on all-cause mortality has, to our knowledge, not been previously investigated in nutrient or food based substitution analyses. In contrast to smaller dietary trials, our findings did not point to any clear potential effect heterogeneity of this particular genotype for any PUFA-rich foods [13–15]. Although the substitution of SFA with carbohydrates was associated with all-cause mortality in men carrying the CT/CC-genotype and not the TT-genotype, these findings may be explained by lower statistical power in the TT-strata, as indicated by the wide CI. Interestingly though, we did observe some stratum-specific results for the addition of SFA on all-cause mortality, with a stronger association in men carrying the CT/CC-genotype compared to the TT-genotype [HR 1.44 (95% CI 1.05, 1.07)] versus [HR 0.98 (95% CI 0.66, 1.44)]. This is to our knowledge a new observation with potential implications for personalized nutrition, if confirmed in other cohorts. Whether these findings may be explained by differences in the distribution of metabolic risk factors (and thereby potential interactions with dietary factors), as indicated by other studies [14, 39], is highly speculative. RCTs by which SFA-rich foods, such as butter, is replaced by PUFA-rich or other macronutrient-rich foods or added to the habitual diet may provide a deeper understanding of this specific gene-diet interaction on cardiometabolic risk markers.

There are several strengths and limitations worthy to mention. First, since the ULSAM cohort has collected unique phenotype characteristics and multiple measurements among these elderly participants, the sample size is relatively small, i.e. the statistical power to detect associations for CVD might have been compromised. Thus, the results should therefore be interpreted with caution, as outlined in the discussion above. Due to this, CVD as an outcome was determined to be secondary to all-cause mortality. Secondly, the study population consisted of only men, which may limit transportability to other populations including women. Likewise, due to potential effect measure modification by the rs174550 FADS1 genotype for the association of SFA on all-cause mortality, different SNP distributions may provide somewhat different estimates in other populations. Furthermore, since pooled nutrients such as SFA, PUFA and carbohydrates may reflect different foods (and/or subclasses of the pooled nutrients) over different age spans as well as geographical locations, this may further impact the extent to which extrapolations of our findings can be done. However, using specified substitution analyses may indeed help mitigate some of the issues of generalizability and transportability imposed by traditional nutritional epidemiological approaches that adjust for total energy intake without any clear replacement foods or nutrients in mind [40]. Additionally, all covariates (including diet) were self-reported which may have led to measurement error and thereby measurement bias/residual confounding bias. Using mainly foods instead of nutrients as the exposure and adjusting for total energy intake in combination with other foods/nutrients might have reduced some of this potential bias [41]. There are however two potential caveats with food substitution models that are important to bear in mind, namely (1) the practical implications of each replacement (100 kcal of meat corresponds to 70 g whereas 100 kcal of F&V corresponds to 200–250 g) and (2) the inherent restriction in the underlying dietary pattern that is imposed by adjusting for all other foods in the model. However, we do believe that the former caveat is feasible for some individuals and the latter of adjusting for all other foods is necessary to answer the research question of isolated food substitutions. Lastly, diet was only measured at one point in time, treating diet as a time-fixed exposure. Further analyses should be conducted with repeated measures of diet (and confounders) to allow for changes over time with the use of more appropriate statistical methods (i.e. g-methods) to account for exposure-confounding feedback when conditioning on post-baseline covariates influenced by past exposure. Strengths of this study include the long follow-up to detect associations for all-cause mortality, the use of a 7-day food record to capture detailed information on the exposure, the use of DAGs to identify potential confounding paths, no loss to follow-up due to the ability to link individuals to different registries through their personal identification number, and the use of substitution (and addition) models to potentially mimic real-life dietary choices.

In conclusion, regarding the replacement of foods varying in fat quality, few associations were observed for the food groups we investigated and all-cause mortality and CVD in this population. These findings might suggest that in Swedish elderly men, replacements of single foods varying in fat quality alone may not be sufficient to counteract other age-related biological processes associated with these outcomes. However, substituting SFA with carbohydrates was associated with decreased all-cause mortality and adding SFA to the habitual diet in men with the CT/CC genotype in the FADS1 gene (rs174550) was positively associated with increased mortality. This association is to our knowledge novel and might point towards a gene-diet interaction with potential clinical implications. Our findings warrant further investigation and confirmation in larger population-based studies.

### Supplementary Information

Below is the link to the electronic supplementary material.

Supplementary file1 (PDF 534 KB)

## Data Availability

Data described in the manuscript will be made available upon reasonable request from the corresponding author.
