# Supplementary material for: Substitution analyses of foods with varying fat quality and the associations with all-cause mortality and impact of the FADS-1 genotype in elderly men
Source: Eur J Nutr. 2023 Sep 20;63(1):145–53. doi: 10.1007/s00394-023-03249-y (PMC10799108; doi:10.1007/s00394-023-03249-y)
Supplement: Supplementary file 1 — Supplementary file1 (PDF 534 KB) [file 394_2023_3249_MOESM1_ESM.pdf]

Substitution analyses of foods with varying fat quality and the associations with all-cause mortality and impact of the FADS-1 genotype in elderly men

Supplementary material for the European Journal of Nutrition

Fridén M[1], Olsson E[2], Lind L[3], Rosqvist F[1], Risérus U[1]

[1]Department of Public Health and Caring Sciences, Clinical Nutrition and Metabolism, Uppsala University, Uppsala, Sweden.

[2]Department of Surgical Sciences, Medical epidemiology, Uppsala University, Sweden.

[3]Department of Medical Sciences, Clinical Epidemiology, Uppsala University, Sweden.

Corresponding author: Ulf Risérus (ulf.riserus@pubcare.uu.se)

Supplementary Table 1. Baseline dietary intake stratified by the rs174550 FADS1 genotype[a].

	**All-cause mortality (n=1084)**		**CVD (n=932)**	
	**TT**	**CT/CC**	**TT**	**CT/CC**
Total energy intake (kcal/d)	1736 (602)	1694 (585)	1749 (607)	1694 (577)
Butter and butter-based spreads (kcal/d)	67 (107)	60 (102)	68 (109)	60 (109)
Margarine and vegetable oils (kcal/d)	0 (52)	0 (71)	0 (51)	0 (71)
Unprocessed fish (kcal/d)	0 (6)	0 (5)	0 (8)	0 (3)
Red and processed red meat (kcal/d)	135 (99)	132 (87)	136 (99)	132 (87)
SFA (kcal/d)	257 (117)	254 (127)	258 (124)	255 (126)
PUFA (kcal/d)	87 (41)	83 (38)	88 (39)	83 (38)

[a]Data are presented in medians (IQR).

CVD, Cardiovascular disease; FADS1, fatty acid desaturase enzyme 1; PUFA, polyunsaturated fatty acids; SFA, saturated fatty acids.

Supplementary Table 2. Associations between food and nutrient substitutions and additions and all-cause mortality and CVD for complete cases (n=1042)[a].

	All-cause mortality	CVD
**Leave-one-out model**		
SFA with PUFA	1.23 (0.82, 1.85)	1.55 (0.92, 2.61)
Butter with oils	0.94 (0.84, 1.04)	0.89 (0.78, 1.01)
Meat with fish	1.26 (0.80, 1.98)	1.21 (0.68, 2.14)
SFA with carbohydrates	0.75 (0.60, 0.93)	0.89 (0.67, 1.18)
Butter with F&V	0.88 (0.75, 1.02)	0.93 (0.77, 1.12)
Meat with F&V	1.01 (0.87, 1.18)	1.11 (0.92, 1.33)
**The all-components model**		
SFA	1.19 (0.93, 1.53)	1.03 (0.75, 1.42)
PUFA	1.48 (0.88, 2.48)	1.61 (0.86, 3.02)
Butter	1.11 (0.99, 1.25)	1.08 (0.94, 1.25)
Oils	1.04 (0.97, 1.12)	0.97 (0.87, 1.07)
Fish	1.22 (0.78, 1.89)	1.11 (0.63, 1.93)
Meat	0.96 (0.86, 1.07)	0.91 (0.80, 1.04)

[a]Data are presented as hazard ratios with 95% confidence intervals.

For complete-case analyses, participants with missing data on covariates were excluded (n=91). Covariates with missing data were: physical activity, smoking, family history of T2D, family history of CVD, sleep and stress.

Both models are adjusted for age, education, smoking, stress, sleep, family history of CVD, family history of type-2 diabetes and physical activity. The leave-one-out model is furthermore

adjusted for total energy intake and includes all nutrients or foods except for the one to be substituted. The all-components model includes all nutrients or foods. All substitutions and additions are modelled in the unit of 100 kcal.

Supplementary Table 3. Associations between food and nutrient substitutions and additions and all-cause mortality and CVD, stratified by rs174550 FADS1-genotype for complete cases (n=1042)[a].

	**All-cause mortality**		**CVD**	
	**TT**	**CT/CC**	**TT**	**CT/CC**
**Leave-one-out model**				
SFA with PUFA	1.37	0.99	1.28	1.37
	(0.73, 2.59)	(0.56, 1.76)	(0.56, 2.93)	(0.68, 2.78)
Butter with oils	0.90	0.98	0.91	0.95
	(0.77, 1.06)	(0.84, 1.13)	(0.74, 1.13)	(0.80, 1.15)
Meat with fish	1.25	1.35	1.56	1.20
	(0.59, 2.62)	(0.73, 2.50)	(0.64, 3.84)	(0.53, 2.71)
SFA with carbohydrates	0.70	0.70	0.77	0.91
	(0.49, 1.00)	(0.52, 0.95)	(0.50, 1.18)	(0.61, 1.34)
Butter with F&V	0.81	0.92	0.85	1.06
	(0.64, 1.04)	(0.74, 1.16)	(0.63, 1.16)	(0.81, 1.40)
Meat with F&V	0.92	1.04	0.95	1.22
	(0.72, 1.17)	(0.84, 1.30)	(0.70, 1.30)	(0.93, 1.58)
**The all-components model**				
SFA	1.08	1.41	1.19	1.07
	(0.72, 1.64)	(1.00, 1.98)	(0.71, 1.98)	(0.69, 1.65)
PUFA	1.45	1.47	1.56	1.47
	(0.64, 3.30)	(0.72, 2.98)	(0.55, 4.40)	(0.63, 3.42)

Butter	1.12	1.11	1.02	1.04
	(0.93, 1.35)	(0.94, 1.30)	(0.81, 1.29)	(0.85, 1.28)
Oils	1.01	1.08	0.93	1.00
	(0.89, 1.15)	(0.98, 1.18)	(0.78, 1.12)	(0.88, 1.14)
Fish	1.24	1.32	1.43	1.10
	(0.61, 2.53)	(0.72, 2.43)	(0.60, 3.38)	(0.49, 2.45)
Meat	0.99	0.98	0.91	0.91
	(0.84, 1.17)	(0.84, 1.14)	(0.73, 1.14)	(0.76, 1.09)

[a]Data are presented as hazard ratios with 95% confidence intervals.

For complete-case analyses, participants with missing data on covariates were excluded (n=91). Covariates with missing data were: physical activity, smoking, family history of T2D, family history of CVD, sleep and stress.

Both models are adjusted for age, education, smoking, stress, sleep, family history of CVD, family history of type-2 diabetes and physical activity. The leave-one-out model is furthermore adjusted for total energy intake and includes all nutrients or foods except for the one to be substituted. The all-components model includes all nutrients or foods. All substitutions and additions are modelled in the unit of 100 kcal.

Supplementary Table 4. Associations between food and nutrient substitutions and additions and all-cause mortality and CVD with the first two years of follow up excluded[a].

	**All-cause mortality**	**CVD**
**Leave-one-out model**		
SFA with PUFA	1.25 (0.84, 1.86)	1.29 (0.76, 2.18)
Butter with oils	0.93 (0.85, 1.03)	0.94 (0.82, 1.07)
Meat with fish	1.29 (0.83, 2.00)	1.16 (0.65, 2.07)
SFA with carbohydrates	0.79 (0.64, 0.97)	0.85 (0.65, 1.12)
Butter with F&V	0.89 (0.76, 1.03)	0.96 (0.79, 1.16)
Meat with F&V	1.00 (0.86, 1.15)	1.02 (0.85, 1.24)
**The all-components model**		
SFA	1.19 (0.94, 1.50)	1.11 (0.82, 1.52)
PUFA	1.51 (0.92, 2.48)	1.46 (0.78, 2.75)
Butter	1.11 (0.99, 1.24)	1.03 (0.89, 1.19)
Oils	1.03 (0.96, 1.11)	0.97 (0.87, 1.07)
Fish	1.26 (0.82, 1.94)	1.12 (0.63, 1.97)
Meat	0.98 (0.88, 1.09)	0.96 (0.84, 1.10)

[a]Data are presented as hazard ratios with 95% confidence intervals for all-cause mortality (n=1112) and CVD (n=917).

Both models are adjusted for age, education, smoking, stress, sleep, family history of CVD, family history of type-2 diabetes and physical activity. The leave-one-out model is furthermore adjusted for total energy intake and includes all nutrients or foods except for the one to be

substituted. The all-components model includes all nutrients or foods. All substitutions and additions are modelled in the unit of 100 kcal.

Supplementary Table 5. Associations between food and nutrient substitutions and additions and all-cause mortality and CVD, stratified by rs174550 FADS1-genotype, with the first two years of follow up excluded[a].

	All-cause mortality		CVD	
	**TT**	**CT/CC**	**TT**	**CT/CC**
**Leave-one-out model**				
SFA with PUFA	1.60	0.94	1.34	1.19
	(0.86, 2.95)	(0.55, 1.63)	(0.58, 3.09)	(0.59, 2.40)
Butter with oils	0.87	0.99	0.90	0.99
	(0.74, 1.02)	(0.86, 1.14)	(0.73, 1.11)	(0.83, 1.17)
Meat with fish	1.21	1.39	1.40	1.22
	(0.60, 2.46)	(0.76, 2.51)	(0.56, 3.49)	(0.55, 2.73)
SFA with carbohydrates	0.80	0.75	0.83	0.89
	(0.58, 1.12)	(0.57, 1.00)	(0.55, 1.27)	(0.62, 1.28)
Butter with F&V	0.83	0.97	0.84	1.05
	(0.66, 1.05)	(0.88, 1.20)	(0.62, 1.13)	(0.80, 1.38)
Meat with F&V	0.91	1.05	0.89	1.09
	(0.72, 1.14)	(0.85, 1.29)	(0.66, 1.20)	(0.83, 1.42)
**The all-components model**				
SFA	0.95	1.40	1.03	1.17
	(0.64, 1.40)	(1.03, 1.92)	(0.62, 1.69)	(0.78, 1.75)
PUFA	1.48	1.41	1.38	1.42
	(0.68, 3.21)	(0.72, 2.76)	(0.49, 3.85)	(0.62, 3.25)

Butter	1.10	1.09	1.04	1.03
	(0.92, 1.32)	(0.83, 1.27)	(0.82, 1.31)	(0.85, 1.25)
Oils	0.96	1.08	0.93	1.02
	(0.84, 1.09)	(0.98, 1.18)	(0.78, 1.12)	(0.90, 1.16)
Fish	1.22	1.40	1.36	1.22
	(0.62, 2.40)	(0.78, 2.51)	(0.56, 3.29)	(0.56, 2.70)
Meat	1.00	1.01	0.97	1.00
	(0.86, 1.18)	(0.87, 1.17)	(0.79, 1.20)	(0.83, 1.20)

Data are presented as hazard ratios with 95% confidence intervals for all-cause mortality (n=1075) and CVD (n=891).

Both models are adjusted for age, education, smoking, stress, sleep, family history of CVD, family history of type-2 diabetes and physical activity. The leave-one-out model is furthermore adjusted for total energy intake and includes all nutrients or foods except for the one to be substituted. The all-components model includes all nutrients or foods. All substitutions and additions are modelled in the unit of 100 kcal.

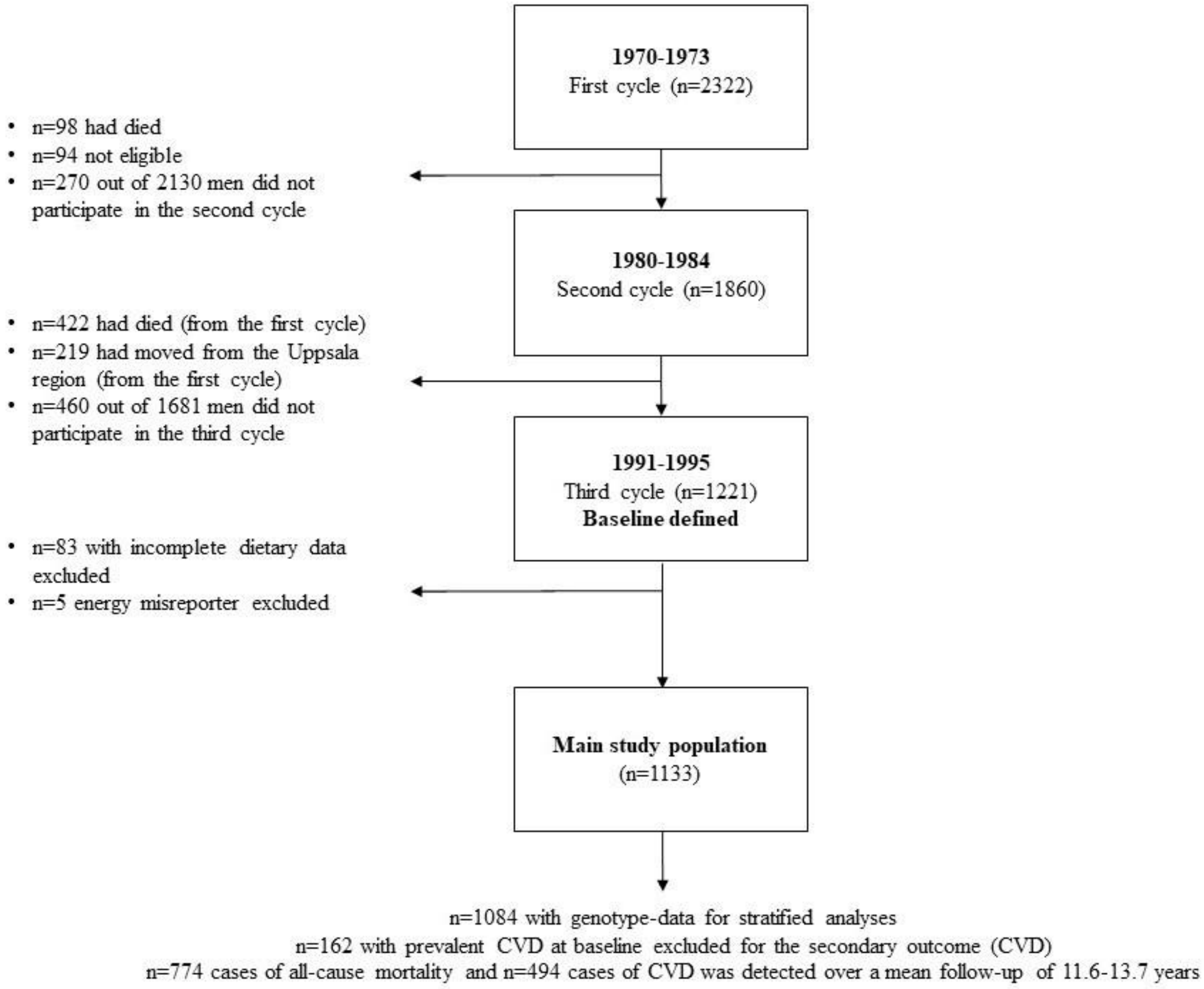

Supplementary Figure 1. Flow-chart of the study.

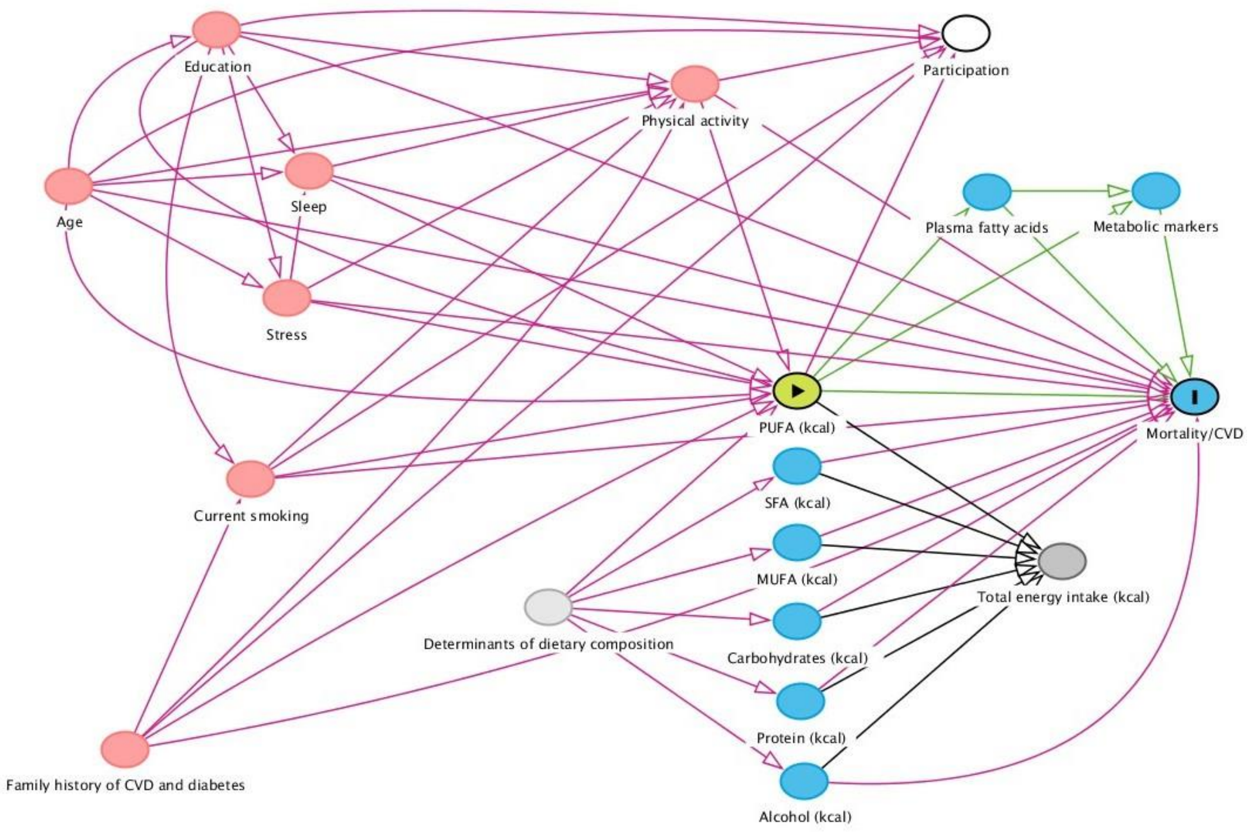

Supplementary Figure 2. Directed acyclic graph (DAG) depicting our assumptions of the causal directions of included variables.

To estimate the joint effect of increasing one nutrient/food while decreasing another, holding all other nutrients/foods fixed, a minimally sufficient adjustment set was identified consisting of family history of CVD, family history of diabetes, current smoking, stress, sleep, education, age, physical activity and total energy intake. These were identified using the Dagitty tool (Dagitty.net). Total energy intake was included to account for confounding by common determinants of dietary composition. The above example includes substituting SFA with PUFA (**leave-one-out model**). In order to estimate the joint substitution effect of increasing PUFA while decreasing SFA, carbohydrates, alcohol, MUFA and protein were additionally adjusted for, leaving SFA out. The same principle was applied to food substitution models, whereby the

composite variable “Total energy intake (kcal)” was subdivided into 10 predefined food categories: meat, fish, butter, vegetable oils, fruits and vegetables, alcoholic beverages, non-alcoholic beverages, total dairy, fatty snacks and pastries and other foods. To estimate the total effect of adding a nutrient or food on top of the background diet (**the all-components model**), the same adjustment set was identified, except for total energy intake. All other nutrients/foods were additionally adjusted for.
